# Perioperative Considerations for Alpha-Gal Syndrome: A Literature Review and Single-Center Experience in a Highly Endemic Area

**DOI:** 10.3390/diseases14080282

**Published:** 2026-08-06

**Authors:** Seth Greenspan, Michele Branigan, Naomi Nguyen, Saba Gulzar, Stephen Probst, Meng Wang

**Affiliations:** 1 Department of Anesthesiology, Renaissance School of Medicine at Stony Brook University, Stony Brook, NY 11794, USA; michele.branigan@stonybrookmedicine.edu (M.B.); naomi.nguyen@stonybrookmedicine.edu (N.N.); stephen.probst@stonybrookmedicine.edu (S.P.); 2 Renaissance School of Medicine at Stony Brook University, Stony Brook, NY 11794, USA; saba.gulzar@stonybrookmedicine.edu

**Keywords:** alpha-gal syndrome, perioperative, anesthesiology, red meat allergy, tick-borne diseases

## Abstract

Alpha-gal syndrome (AGS) is an acquired allergy characterized as a delayed hypersensitivity reaction to the galactose-alpha-1,3-galactose carbohydrate, which is present on mammalian meat. AGS can cause severe anaphylactic reactions and has been implicated in intraoperative reactions to medications or surgical products that are derived from mammalian meat. This narrative review will describe the current literature on alpha-gal etiology, epidemiology, and perioperative management. Additionally, our institution is present in Suffolk County, New York, US, a highly endemic area, and we share our centers’ recommendations for managing AGS patients from their initial preoperative presentation throughout the perioperative period. We propose a detailed pathway for detection, preparedness, and intraoperative management of AGS patients undergoing elective surgery as well as alternative management suggestions for AGS patients undergoing emergency surgery.

## 1. Introduction

The alpha-gal syndrome (AGS) is caused by an acquired hypersensitivity reaction to the galactose-alpha-1,3-galactose carbohydrate which is found in non-primate mammalian tissue. Patients with the syndrome typically must avoid eating “red” meats such as beef, pork, and lamb as well as their innards and organs [1,2]. AGS is more common in regions with high populations of the lone star tick, Amblyomma americanum, whose bite causes alpha-gal sensitization throughout the United States (US) [3].

AGS responses to mammalian derived products include pruritis, angioedema, hives, and anaphylaxis which typically occur 3 to 8 h after eating meat. Reactions can also include gastrointestinal symptoms such as diarrhea, abdominal cramping, or emesis with complete exclusion of cardiovascular or respiratory manifestations [2]. Management in day-to-day life involves avoiding foods and products that contain alpha-gal and preventing tick bites [3]. Unlike most food allergies, AGS reactions are often delayed 3–6 h after exposure, and patients have frequently tolerated red meat for many years prior to the development of allergic reactions [2]. Approximately 20% of patients will also have reactions to alpha-gal content in dairy products, particularly with soft cheese, heavy cream, and ice cream [1].

When consuming dietary sources of alpha-gal, patients typically exhibit a delayed presentation of hypersensitivity reaction with an onset several hours after the ingestion, whereas when injected with alpha-gal-containing medications, patients have a more immediate reaction [1]. For medication reactions, the timing of the alpha-gal reaction similarly correlates with the route of administration of the allergen. Parenteral medications containing alpha-gal typically have an immediate onset of symptoms within 1 h, whereas with oral consumption, the reaction occurs in 3–6 h [4].

Many medications commonly utilized in the perioperative setting are derived from or contain contaminants from mammalian tissue such as heparin and surgical hemostatic agents [5]. While rare, there are several cases of intraoperative anaphylaxis that have been associated with alpha-gal syndrome [6,7]. This risk of severe allergic reaction led clinicians to develop perioperative recommendations for the management of AGS patients in high-risk areas. Dunkman et al. emphasize the institutional recognition of alpha-gal-containing medications/surgical products, use of the electronic medical record (EMR) to document AGS as an allergy, and provider education on the increasing prevalence of AGS near their institution in North Carolina [8]. Nourian et al. describe the most common anesthetic medications and surgical products that can be alpha-gal allergens, recommendations to anesthesia providers to mitigate allergen exposure, and also propose a specific protocol for patients with AGS undergoing cardiovascular surgery utilizing unfractionated heparin [4].

This paper proposes the implementation of a structured preoperative protocol to improve the identification and surgical management of patients with AGS. An example protocol has been developed and utilized within our institution, which is in Suffolk County, NY, a county with one of the highest prevalences of alpha-gal syndrome in the US [9]. Our protocol emphasizes preoperative screening, risk assessment, and multidisciplinary operating room preparedness, and includes discussion of high-risk surgeries including cardiac and neurosurgical procedures.

## 2. Etiology and Pathophysiology of Alpha-Gal Syndrome

The development of AGS sensitization in humans is attributed to tick bites, specifically the lone star tick (*Amblyomma americanum*) as the main vector in the United States [3]. Other ticks that have been known to cause sensitization to alpha-gal are various species of Ixodes ticks (*hococyclus*, *australiensis*, *ricinus*), *Rhipicephalus bursa*, *Haemaphysalis longicornis*, and other *Amblyomma* species (*testudinarium*, *sculptum*, *variegatum*, *herbraeum*) [3]. Studies have shown that alpha-gal is expressed in the saliva of tick species linked with alpha-gal syndrome, though it is not entirely known how the tick acquires and presents alpha-gal for the host to develop anti-alpha-gal IgE [10]. It is possible that alpha-gal antigens may be residual or recycled mammalian glycoproteins or glycolipids from previous blood meals or may be contributed by tick-acquired viruses, protozoans, or bacteria. There is also evidence that alpha-gal possibly originates from the tick itself [11]. Several studies show that tick-associated factors that contribute towards AGS are divided into two categories (1) intrinsic factors and (2) extrinsic factors. Intrinsic factors include tick microbiome, tick glycosylation machinery, host-seeking, and feeding behaviors. Extrinsic factors include geographical distribution of ticks and tick–predator interactions which in turn can limit tick population [11].

It is not entirely understood how ticks are so effective at eliciting IgE, though it is suggested that there is either a unique component to transmission via skin or there is another feature of tick saliva that favors IgE class switch [10]. The connection between tick bites and IgE can be partially explained by considering the role Type 2 immune cells, such as basophils, eosinophils, IgE, histamine, and mast cells, play in defending mammalian hosts against ticks. Studies report that subcutaneous inoculation of mice with the larval stage of the lone star tick extracts promoted IgE class switch that depended on MyD88 signaling in B cells [12]. It is also reported that tick saliva has a high concentration of Prostaglandin E2 (PGE2) which is involved in the reduction and recruitment of macrophages [11]. This in turn drives immune response towards TH2, which plays a primary role in mediating allergic reactions [11]. It has also been shown that PGE2 can directly induce class switching of specific B cells to produce IgE, which is a key factor of allergic responses following antigen exposure [11]. Various other components of tick saliva such as prostaglandins, sphingomyelinase, and a cysteine protease inhibitor are known to induce TH2 profile which is a key factor in shaping the innate immune response [11].

The exact pathogenesis of AGS in humans is not well understood, but it is known to involve IgE-mediated activation of mast cells after mammalian meat is consumed which contributes to the gastrointestinal symptoms of AGS [3]. Oligosaccharide galactose alpha 1,3-galactose (alpha-gal) is a lipid bound carbohydrate allergen that has a slower absorption rate in comparison to typical protein allergens which have quicker absorption and reaction onset [2]. The “glycolipid hypothesis” is well known and describes the mechanism for the delay in response to ingestion of meat products and later development of symptoms. It is thought that after those affected by alpha-gal consume mammalian meat or its derivatives, lipid micelles are formed from glycolipids containing alpha-gal [3]. Enzymes in the small intestine then break down triglycerides into free fatty acids, monoglycerides, and diglycerides which are then absorbed by intestinal cells. Once bundled into chylomicrons that display alpha-gal molecules, these chylomicrons enter the lymphatic system about 4 h after a meal and the alpha-gal molecules bind to alpha-gal specific IgE antibodies on the surface of basophils or mast cells [3]. This ultimately triggers an allergic reaction, such as hives or pruritus, or a more severe systemic reaction, such as anaphylaxis, as mast cells release allergic mediators which also stimulate sensory nerves, causing intestinal smooth muscle contraction resulting in abdominal visceral pain and excessive mucous secretion [3].

As a result, there are a variety of mammalian products that contain higher levels of alpha-gal that should be avoided by those with AGS. In a study testing IgE reactivity among several different mammalian meats and products, it was found that the highest reactivity occurs against pork innards and beef kidney [13]. Lean meats, such as reindeer, moose, beef, and wild boar, and processed meats, such as bacon and chorizo, showed moderate reactivity [13]. IgE reactivity to liver pâté and whey was low while milk and “black pudding” (a blood sausage) were the lowest [13]. While gelatin was not included in this study, it has been known to cause reactions in those with AGS as well [14]. These reactions overall correlated with the levels of alpha-gal in the listed mammalian products. Additionally, patients with higher alpha-gal-specific IgE levels show a higher IgE binding to food extracts [13]. The blood level of IgE for alpha-gal often decreases over time in patients who avoid recurrent tick bites although the rate of the decline is variable [15]. Future exposure to tick bites can further augment the IgE antibody response [16].

AGS reactions are typically inconsistent, and progression to more consistent reactions is typically suggestive of a new tick bite. AGS reactions can also exclusively affect the gastrointestinal tract as many of these patients were originally diagnosed with chronic diarrhea, IBS, or gastrointestinal food allergy syndrome prior to AGS diagnosis [2]. These symptoms typically resolve following diagnosis and proper avoidance of food triggers, while oral cromolyn is useful in those with continuing symptoms [2].

## 3. Epidemiology of Alpha-Gal Syndrome

Alpha-gal syndrome was first observed in the United States (US) in the mid-2000s as a rapid-onset anaphylactic reaction to cetuximab infusions among patients previously exposed to galactose-α-1,3-galactose [17,18]. Regional variations in cetuximab hypersensitivity reactions suggested an environmental or acquired cause of the reactions, as more frequent cases were observed in the southeastern states such as Tennessee and North Carolina [19]. Also in the mid-2000s, an association between tick bites and acquired red meat allergy was discovered in Eastern Australia [20]. The increased incidence of AGS in the US over the past two decades has been linked in part to an increase in the population and range of *Amblyomma americanum* which is the most common tick found on humans in the United States and responsible for most AGS sensitization [21,22,23]. While the tick species is consolidated in the Southern United States, tick-borne illnesses associated with *Amblyomma americanum* have been diagnosed as far north as southern New York and New England [22].

Alpha-gal syndrome has now been diagnosed internationally, with cases in Europe, Central and South America, Asia, and Australia, although the causative tick is not exclusively *Amblyomma americanum* [15]. AGS cases identified in Australia have been attributed to *Ixodes holocyclus*, while the syndrome has been acquired in Europe due to the *Ixodes ricinus* tick [24,25]. In Asia, *Haemaphysalis longicornis* and *Amblyomma testudinarium* have been implicated, while *Amblyomma scupltum* has been associated with AGS in South America [25,26]. Global estimates of the prevalence of AGS are difficult to determine, although it is likely to be increasing due to the increased range and population of tick species [25,27]. A study from Spain found a prevalence of 15% positivity of serum IgE to alpha-gal in a population of foresters with high risk of tick bites, while only 4% positivity in a control group [28]. A study of 300 German foresters found 35% had elevated levels of serum alpha-gal IgE although only 2% of the total cohort were symptomatic [29]. A 2015 study estimated the prevalence of syndromic AGS in Australia as 1/550 [30].

There is substantial evidence the prevalence of AGS has been increasing throughout the United States, although with significant geographic variations [9,31,32]. A retrospective analysis of all alpha-gal IgE serum samples collected from 2010 to 2018, found that more than 34,000 positive tests were reported from 122,068 patient samples (32% positive rate), with a 6-fold increase in positive tests from 2010 to 2018 [32]. A majority of samples in this study were collected from the South (68%), with the fewest from the West (2%); the positivity rate of samples was similar across the South, Northeast, and Midwest (~30–35%) and lowest in the West (7%) [32]. However, from 2017 to 2022, 357,119 samples on 295,400 patients were submitted with 90,018 people testing positive (31%), which was a similar positivity rate to that observed between 2010 and 2018, although the overall incidence increased [9]. Similarly, most samples were submitted from states in the South, Midwest, and Mid-Atlantic [9]. Alpha-gal serum positive patients tend to be older (mean age 48 in the positive group versus mean age 41 in the negative group), although children as young as 4 have been reported to have AGS [9,33].

The rate of positive serum tests for IgE antibodies may overrepresent the incidence of AGS as many seropositive patients do not clinically display symptoms of mammalian meat allergy. A cross-sectional study of 404 patients undergoing screening colonoscopy at a single center identified 31% of patients as positive for alpha-gal IgE antibodies [34]. However, when comparing patients sensitized to alpha-gal to those without antibodies, the researchers found the same rate of abdominal pain and mammalian meat consumption between groups [34]. A retrospective study examined a large electronic health record including data on over 114 million US patients and found only 180 diagnoses of mammalian meat allergy between 2015 and 2020, although with a 5520% increase to 10,132 cases between 2021 and 2025 [31]. In regions with endemic ticks, sensitization rates can be as high as 15–30%, although only 1–8% of sensitized individuals will display signs of clinical alpha-gal syndrome [2,29].

## 4. Diagnosis

The presentation of AGS is highly variable which makes accurate diagnosis difficult for clinicians. Some clinical characteristics suggestive of AGS include onset in adulthood after previous ability to tolerate mammalian meat, hypersensitivity reactions (pruritis, angioedema, urticaria or anaphylaxis) or gastrointestinal symptoms (diarrhea, abdominal cramping, emesis), delayed onset 3–8 h after eating mammalian animal product, clinical improvement when abstaining from mammalian food consumption, and reported history of tick bites [2,5]. Some experts suggest alpha-gal syndrome should be considered in the differential diagnosis for any patient with recurrent episodes of urticaria or anaphylaxis who live in a region with endemic *Amblyomma americanum* ticks [10].

In patients with clinical suspicion for AGS, serum alpha-gal IgE testing is recommended to confirm the diagnosis [2,10]. Commins describes a diagnostic algorithm for alpha-gal syndrome, including when to recommend testing based on clinical presentation [2]. Serum testing is offered by Viracor-Eurofins in the US and ThermoFischer Scientific in Europe, which use immunoassays to test for IgE levels in the serum sample against the single antigen galactose-alpha-1,3 galactose [2]. There is no clear serum IgE positive cutoff value that optimizes sensitivity and specificity for diagnosis of AGS, however values of 0.1 IU/mL, 0.35 IU/mL, 0.59 IU/mL, and 2 IU/mL have been suggested [32,35,36,37,38]. Conventional skin prick tests for allergens to beef, pork, or lamb do not reliably diagnose alpha-gal syndrome [39]. Additionally, clinicians should be aware of other potential meat allergies such as pork–cat syndrome that are not associated with elevations in alpha-gal IgE as acquired from a tick bite [2].

## 5. Alpha-Gal Syndrome and Medication Allergies

Pharmaceutical/medical products implicated in cases of alpha-gal-mediated hypersensitivity reactions include monoclonal antibodies, gelatin-containing medications, pancreatic enzymes, hemostatic agents, bovine or porcine prosthetic heart valves, antivenom, heparin, and magnesium stearate [5]. Biologic mesh, catgut suture, surgical hemostatic powder, and xenografts can also trigger an immune response [40]. The monoclonal antibody cetuximab, which is partially mouse-derived and contains alpha-gal on its heavy chain, has caused anaphylaxis reactions in patients with alpha-gal syndrome, and there are case-reports of ramucirumab and infliximab causing alpha-gal-mediated reactions as well [26,41,42].

One of the challenges of identifying the cause of an alpha-gal-associated reaction is that the source may be an inactive ingredient such as magnesium stearate or glycerin; however, the US Food and Drug Administration does not require pharmaceutical manufacturers to report changes in inactive ingredients in their products [43]. One alpha-gal-sensitized patient had reactions to acetaminophen, naproxen, lisinopril, hydrocodone/acetaminophen, and clonidine which all share magnesium stearate as an inactive ingredient [43]. Two patients without known allergies had anaphylaxis to Group B FFP transfusion and were subsequently found to have positive alpha-gal IgE antibodies, suggesting a possible correlation [44].

Intraoperative anaphylaxis is rare with an estimated incidence between 1:4000 and 1:25,000, and only a small subset of those cases will be linked to AGS [7]. Most anesthetic drugs used in common practice are safe to use in patients with AGS, although some may contain inactive ingredients that can potentiate an AGS allergic reaction [45]. Some potentially unsafe intravenous medications in common anesthetic practice include propofol (containing glycerol), hydromorphone, clevidipine, milrinone, and vasopressin [45]. Analgesics commonly ordered for postoperative pain such as lidocaine patches, acetaminophen tablets/capsules, other opioids, gabapentinoids, and muscle relaxants can also contain triggering agents [4,45].

Gelatin-based capsules, in general, are commonly used to deliver oral medications. Gelatin itself is used as a colloid plasma expander outside the US and is also present in several vaccines and hemostatic agents [5,46]. The MMR, varicella, and herpes zoster vaccines can contain gelatin and have been implicated in alpha-gal anaphylactic reactions in sensitized patients [47,48]. There is a case report of an intraoperative anaphylactic reaction to Surgiflo (Hemostatic Matrix; Ethicon Inc, Somerville, NJ, USA), which is a hemostatic agent that contains a porcine gelatin matrix [7]. The case involved an AGS patient undergoing an atrial septal defect repair who developed hemodynamic instability after Surgiflo was applied over sutures on his right atrium which resolved with administration of epinephrine [7]. A patient in Spain who later tested positive for IgE antibodies to alpha-gal developed anaphylaxis requiring antihistamine and steroids after receiving intravenous gelafundin, a gelatin-based plasma expander, which was used to treat hypotension after spinal anesthesia [49].

Heparin molecules (both unfractionated and low-molecular weight) do not contain alpha-gal but they are derived from animal products including pig intestines or bovine lung and therefore may be cross-contaminated with alpha-gal in their manufacturing [50]. The use of unfractionated heparin for cardiovascular surgery puts patients at risk of allergic reactions, and intraoperative anaphylaxis has been described [4,51]. Given levels of alpha-gal contamination of heparin can vary by lot, one strategy to avoid intraoperative anaphylaxis with intravenous heparin, such as during cardiac surgery with cardiopulmonary bypass, is to give a test dose of the medication preoperatively from the same vial that will be used in the operating room [50]. An alternative consideration is premedication with glucocorticoids and antihistamines for patients with low levels of IgE to alpha-gal, which was successful for a catheter ablation procedure [52]. Other potentially unsafe anticoagulants include apixaban, rivaroxaban, warfarin, aspirin, clopidogrel, prasugrel, and ticagrelor [45].

Alpha-gal is present on mammalian tissue used in prosthetic heart valves and has been well known to be immunogenic as well as a potential cause of long-term valve degradation [53]. While the alpha-gal IgA, IgG and IgM antibody responses have been well described for decades, the development of IgE antibodies to alpha-gal on mammalian valves in patients with AGS is a novel concern and may increase the risk of adverse cardiovascular outcomes in these patients [54]. There are also many products derived from mammalian tissue that are commonly utilized in complex spine surgery and brain surgery such as hemostatic agents, allografts, dural reconstruction products, and glues [55,56].

## 6. Preoperative Evaluation

The mainstay of perioperative management of alpha-gal syndrome is early detection, avoiding triggering agents, and recognition of the symptoms of a reaction if it occurs. The perioperative detection and management of AGS begins at the preoperative visit. A multidisciplinary approach including allergists, laboratory medicine specialists, pharmacy services, and preoperative clinics is essential in preventing the use of mammalian products in the operating room as well as in the preoperative and postoperative settings [4]. When a patient with a history of mammalian meat allergy and/or tick bite presents to the preoperative clinic, additional screening should occur to assess risk of alpha-gal reactions. If the patient reports an allergic reaction to monoclonal antibodies or gelatin-containing vaccines, suspicion is raised for alpha-gal syndrome.

There is variability in patient sensitivity to alpha-gal such that some individuals can tolerate low levels of exposure with minimal symptoms. A detailed history should be obtained regarding reactions to meat products, gelatin products, milk/dairy, and over-the-counter (OTC) gelcaps/gummies. If the patient experienced a reaction to any of these products, determine the nature and severity of the reaction. Determine if the patient can tolerate gelatin and dairy. Determine if the patient experienced a prior tick exposure. Ascertain if the patient tolerates OTC medications and obtain a list of current prescription medications. Inquire as to when the most recent reaction took place as well as the severity of the reaction. The more oral medications or animal-based food products a patient can tolerate, the less worrisome it is that a severe intraoperative reaction will occur [4]. A risk-versus-benefit analysis of each medication or product should be performed on a case-by-case basis prior to use [8].

When the diagnosis of alpha-gal syndrome is reported at the preoperative visit, the evaluation should include how and when the allergy was diagnosed. Was it confirmed by an allergist? If there is concern for AGS that has not previously been diagnosed, a referral to an allergist should be made and an alpha-gal-specific serum IgE blood test should be performed [4]. Also inquire as to what treatments have been effective for prior symptoms as well as whether the patient has an EpiPen and if it was ever used. Obtain the previous records from the allergist if available regarding past exposures, any progression of allergic response, and previous titers of alpha-gal and IgE.

The allergy profile in the electronic medical record (EMR) should be updated to reflect the alpha-gal allergy as well as a red meat allergy to avoid dietary triggers. The EMR allergy alert will likely only generate an alert for drugs with a known risk, such as heparin or cetuximab, as many systems are not set up to screen for inactive ingredients. Generated lists of agents with mammalian derivatives commonly used perioperatively are dynamic as the content may change at any time. Determine if the patient has taken regular medications from the pharmacy in the past or if special precautions have been utilized. Inactive ingredients in medications vary by manufacturer lot number. The hospital pharmacist can determine inactive ingredients by looking up the National Drug Code (NDC) number and entering it into Daily Med, a US National Institutes of Health sponsored drug database [4].

When AGS is revealed during the preoperative assessment, there must be a clear line of communication between the preoperative clinic, pharmacy, and the anesthesiologist. It is important for the surgeon to be aware of the allergy since mammalian products, such as heparin, are commonly utilized in the operating room. Greater risk of a severe allergic reaction may be related to a higher preoperative alpha-gal titer. It is important to know the IgE level prior to surgery and premedicate the patient as needed for surgeries with high risk of exposures [55,57]. At the preoperative clinic, it may be reasonable to pre-emptively identify outpatient pharmacies that can provide safe postoperative medications [40]. Special attention should be paid to patients enrolled in ERAS (Enhanced Recovery After Surgery) protocols outlining pre-emptive multifocal analgesic medications as part of the ERAS pathway [5].

## 7. Perioperative Allergen Control for Alpha-Gal Syndrome

**Figure 1:**
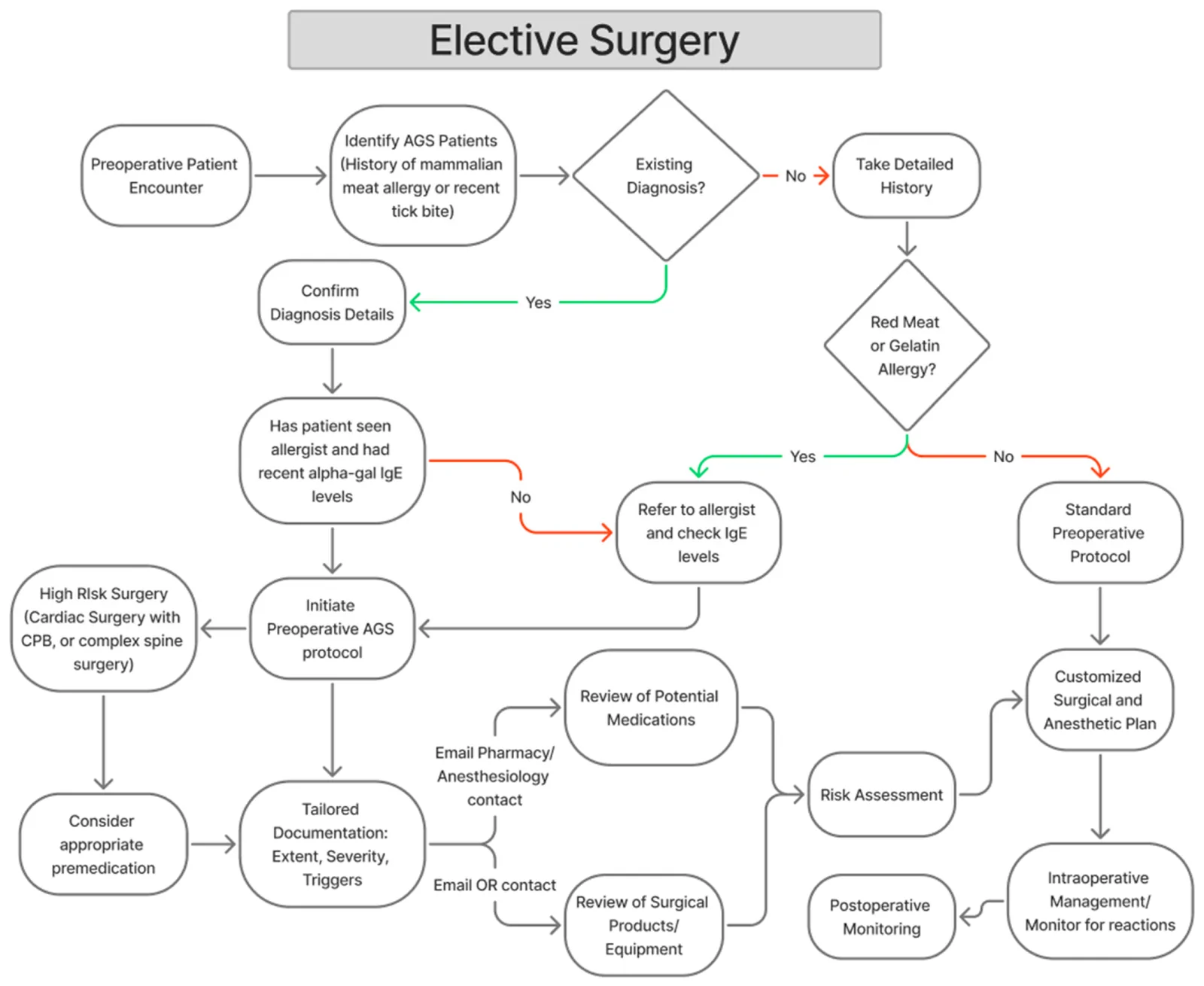
Institutional protocol for perioperative management of patients with alpha-gal syndrome presenting for elective surgery.

An institutional perioperative protocol for AGS should comprise three core phases: identification, preparation, and intraoperative management. While some details of our institutional protocol as well as recommended practices will be described, these should not substitute clinical judgment when treating patients with confirmed or suspected AGS. A flow-chart of this protocol for elective surgery is presented in Figure 1.

The first phase is appropriate identification and risk stratification of patients with AGS, which occurs in the preoperative clinic as discussed in the previous section. Patients with confirmed AGS or those at risk should have “alpha-gal” added to their EMR allergy record, which will allow for allergy banding on the day of surgery. Our preoperative clinic routinely sends a notification to our AGS taskforce which includes the anesthesiologist director of preoperative services, a designated operating room pharmacist familiar with alpha-gal triggering medications, and a representative from operating room management to ensure safe use of surgical supplies and equipment. Additional confirmation of patients with AGS occurs on the day of surgery during the preoperative assessment by surgical, anesthesia, and perioperative nursing teams as well as during the “time-out” in the operating room prior to induction of anesthesia and again before the start of the procedure.

Institutional organization in collaboration with pharmaceutical services is critical in ensuring safe preparation for AGS patients to undergo elective surgery. To ensure optimal care, healthcare institutions should designate dedicated pharmacy groups specifically responsible for developing and maintaining alpha-gal safe anesthesia medication inventory and workstations. One of the challenges of reducing sensitized patients’ exposure to alpha-gal-triggering medications is that pharmaceuticals frequently change their manufacturing process, allowing potential inactive ingredients to be implicated in reactions. Our institution routinely monitors commonly used anesthesia-drug inventories and periodically reviews the associated National Drug Codes (NDCs) to maintain up-to-date accuracy. The NDCs link to a specific drug with both generic and proprietary names, route of delivery, and manufacturer [40]. However, providers should beware that the packaging of some generic medications may only include the lot number from the distributor which could include a mix of different manufacturers, without clear NDC labeling [40]. These potential pitfalls emphasize the importance of pharmacist oversight into a multidisciplinary care structure.

In elective cases with a high index of suspicion, pharmacy review of individual medication batches is recommended but is resource and labor-intensive. In such cases, considerations should be made to balance this effort against the severity of the patient’s allergy as those with limited, mild symptoms and tolerance of a variety of prior medications may have a lower risk of reaction to contaminants than patients with severe, compromising symptoms.

Preoperative preparation must include operating room management to identify medications and materials used by the surgical team that would be contraindicated in AGS patients and offer recommendations for safe alternatives. Some common surgical hemostatic agents derived from mammalian-thrombin that have been flagged as potentially unsafe include Gelfoam (absorbable gelatin compressed sponge, Pfizer, New York, NY, USA), FloSeal (hemostatic matrix, Baxter, Hayward, CA, USA), Surgiflo (hemostatic matrix; Ethicon Inc, Somerville, NJ, USA), Surgifoam (absorbable gelatin, Johnson & Johnson, New Brunswick, NJ, USA), Tisseel (fibrin sealant, Baxter, Deerfield, IL, USA), or Recothrom (topical thrombin, Baxter, Deerfield, IL, USA) [55]. Safer hemostatic agents would be ones made from plant/cellulose-based products such as Surgicel (absorbable hemostat, Johnson & Johnosn, New Brunswick, NJ, USA) or recombinant/human thrombin products [55]. Advanced preparation of the appropriate resources enables the surgical/anesthesia team to efficiently and safely manage AGS cases and make informed, timely decisions regarding the selection of anesthesia agents and operative materials. Critically, this framework depends on early detection and clinical suspicion of AGS at the outset of the perioperative process.

Two notable surgeries with a high risk of alpha-gal-mediated reactions deserve special mention: cardiac surgery requiring cardiopulmonary bypass and complex spine surgery which frequently uses high volumes of mammalian-derived hemostatic products. In either surgery type, exposure to alpha-gal-containing ingredients may not be avoidable, and premedication is considered to lessen an allergic response. Nourian et al. developed a protocol for managing AGS patients requiring procedural parenteral heparin administration, based on serum IgE levels [4]. Patients with higher IgE levels are recommended to have desensitization in the ICU preoperatively, while lower risk patients can undergo premedication and heparin loading challenge, with alternative anticoagulant use (argatroban or bivalirudin) is recommended if any of the above strategies fail or the case is urgent/emergent [4]. Razzaq et al. developed a similar pretreatment protocol for patients undergoing complex spine surgery at high risk of AGS reactions based on IgE levels [55].

**Figure 2:**
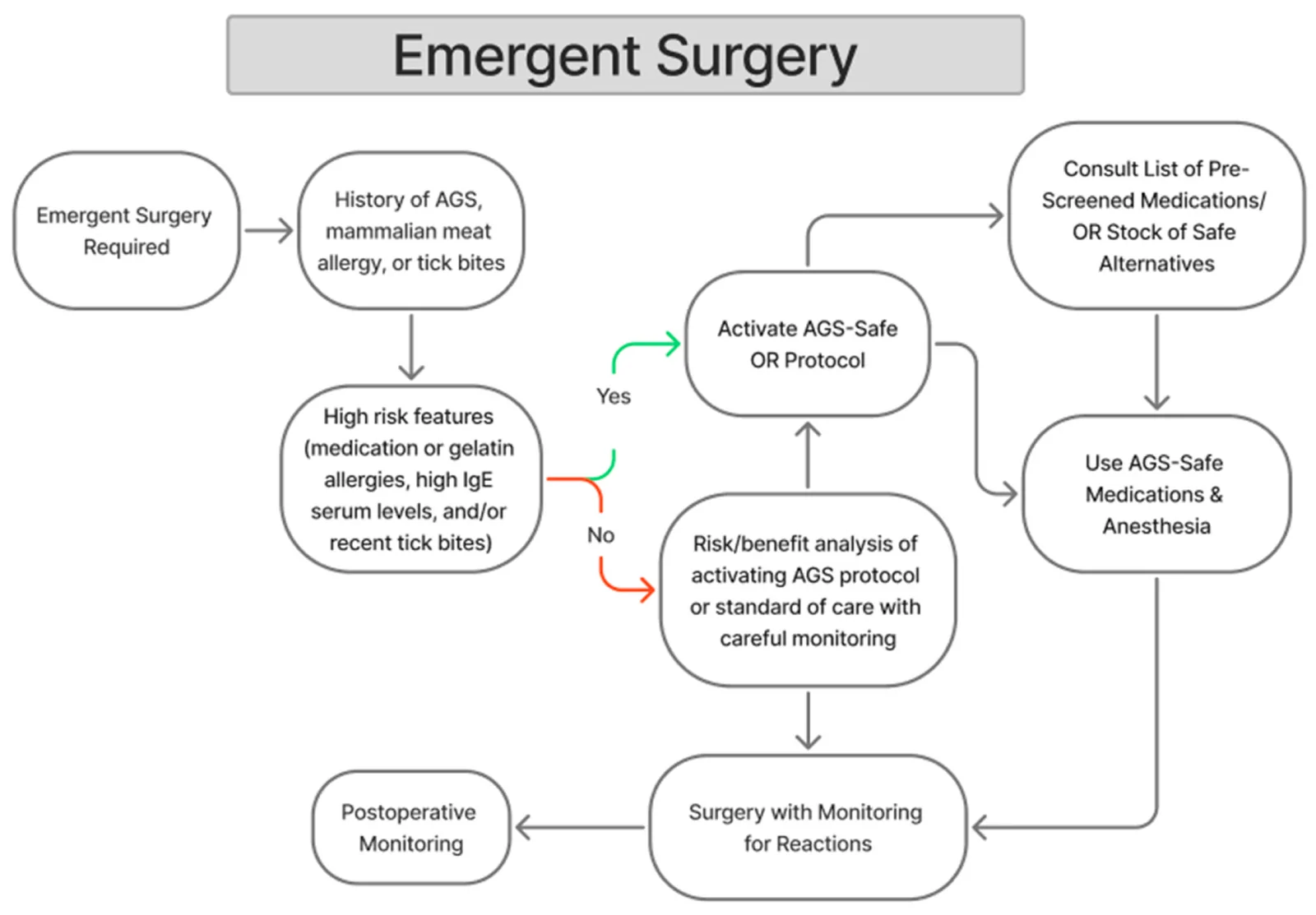
Institutional protocol for perioperative management of patients with alpha-gal syndrome presenting for emergency surgery.

It is important to consider that emergency cases present additional challenges, as limited pharmacist availability may preclude real-time batch verification. This leads to the consideration of two potential institutional strategies that may allow for effective management of AGS patients in emergency surgical situations. Firstly, institutions may adopt the use of pre-screened and assembled “Alpha-gal-safe” packs consisting of essential anesthetic agents. However, these packs require consistent maintenance. This is resource intensive, as pharmacy staff must routinely inspect, restock, monitor expiration dates, and ensure proper storage conditions. This significantly increases the oversight requirement, compromising reliability when these supplies are urgently needed. Secondly, if institutions were to coordinate with designated pharmacy groups to prepare a maintained list of alpha-gal-safe medications, organized by NDCs, this would sufficiently allow for the seamless implementation of alpha-gal-free operating rooms—recognizing that some agents without alternatives remain exceptions. Such management further minimizes uncertainty during emergent care, allowing anesthesiologists to safely manage patient care. A flow-chart of a protocol for AGS patients undergoing emergency surgery is represented in Figure 2.

Anesthesia providers should also be wary about the risk of reactions to medications commonly prescribed in the postoperative period, such as opioids and acetaminophen tablets. Management of these medications requires ongoing multidisciplinary coordination and standardized institutional guidelines to ensure comprehensive safety for AGS patients as they transition from the perioperative setting through their postoperative care.

## 8. Conclusions

Alpha-gal syndrome poses considerable challenges to perioperative clinicians in endemic areas given its increasing prevalence and its variable presentation. Multidisciplinary collaboration between allergy and immunology, anesthesiology, pharmacy, laboratory medicine specialists, and surgery teams is essential for preventing intraoperative alpha-gal-mediated anaphylactic reactions which can be life threatening. The main goal of perioperative management is to identify patients at risk for reactions and minimize the risk of exposure by utilizing safe medications and surgical products. Anesthesiologists, pharmacists, and surgeons must remain vigilant to the ever-changing manufacturing process of various medications and surgical products that may contain mammalian derivatives or contaminants. Future research and initiatives to identify plant-based alternatives and wholly avoid animal-based products in medical care may reduce overall iatrogenic allergic reactions given the increasing prevalence of alpha-gal syndrome.

## Data Availability

No new data were created or analyzed in this study. Data sharing is not applicable to this article.

## Abbreviations

The following abbreviations are used in this manuscript:

AGS	Alpha-gal syndrome
ERAS	Enhanced Recovery After Surgery
EMR	Electronic medical record
ICU	Intensive care unit
OR	Operating room
OTC	Over-the-counter
NDC	National Drug Code
PGE2	Prostaglandin E2
US	United States
